# Longitudinal relationships between serum ferritin and prognosis among severe community-acquired pneumonia patients

**DOI:** 10.3389/fphar.2025.1556185

**Published:** 2025-05-20

**Authors:** Jing Xu, Ming-Feng Han, Kai-Shu Ma, Ting Zhang, Rui-Rui Wang, Jing-Jing Zhang, Hui Zhao, Lin Fu

**Affiliations:** ^1^ Department of Respiratory and Critical Care Medicine, No.2 People’s Hospital of Fuyang City, Fuyang, Anhui, China; ^2^ Fuyang Infectious Disease Clinical Collage of Anhui Medical University, Fuyang, Anhui, China; ^3^ Department of Respiratory and Critical Care Medicine, Funan County People’s Hospital, Fuyang, Anhui, China; ^4^ Department of Respiratory and Critical Care Medicine, Second Affiliated Hospital of Anhui Medical University, Hefei, Anhui, China; ^5^ Institute of Respiratory Diseases, Second Affiliated Hospital of Anhui Medical University, Hefei, Anhui, China

**Keywords:** ferritin, community-acquired pneumonia, severity, prognosis, longitudinal relationship

## Abstract

**Background:**

Ferritin is one of the major intracellular iron storage proteins and is implicated in the pathophysiological processes of many inflammatory diseases, but its function in community-acquired pneumonia (CAP) was unclear. The purpose of this study was to evaluate the expression of ferritin and analyze the relationship of serum ferritin with CAP.

**Methods:**

Severe CAP patients and age- and gender-matched healthy participants were recruited for the study. Serum ferritin was detected through ELISA. Physiological characteristics were recorded.

**Results:**

The serum ferritin level was significantly increased in severe CAP patients upon initial hospitalization compared to healthy participants, and it decreased after therapy. Correlative analyses hinted that there were obvious relationships of serum ferritin with the indicators of blood routine, liver function, and inflammation. Moreover, linear and logistic regression analyses confirmed that the serum ferritin level was positively related to the scores of CURB-65, CRB-65, and PSI. The poor prognosis including mechanical ventilation, vasoactive agent, ICU admission, death, and longer hospital stays were assessed in severe CAP cases during hospitalization. Multivariate logistic regression showed that higher serum ferritin levels were closely linked to the poor prognostic outcomes.

**Conclusion:**

There is significantly positive association between the serum ferritin level upon initial hospitalization with the severity and poor prognosis. Thus, serum ferritin could be an indicator for the determination of severity and prognosis among CAP cases.

## Introduction

Community-acquired pneumonia (CAP) is a frequent cause of acute inflammatory illness in different aged populations and one of the most common reasons of hospital admission. Mounting pieces of evidence have revealed that CAP is strongly associated with mortality and hospitalization across the whole world (Brown and Lerner, 1998; Marrie, 2000). The mortality rate is quite high in hospitalized patients of all ages, ranging from 5% to 15% (Chang et al., 2016). Rapid and reasonable treatment can effectively downregulate the mortality, and earlier discrimination of the state of illness is significant for ameliorating the severity in CAP patients (Jiang et al., 2021; Wang et al., 2021; Feng et al., 2021a). Though there have been great advancements in treatment tools and detection methods, the clinical, radiological, and biological characteristics are minimally sensitive or specific (Christ-Crain and Opal, 2010). Moreover, many microbiological detection methods often are slow and invalid. Hence, new indicators might assist in evaluating the illness among CAP cases.

Iron is essential for survival and reproduction in nearly all life forms. Iron application can affect the growth and pathogenic potential for microbes (Ganz and Nemeth, 2015). Various defense mechanisms have gradually been formed in the bodies’ immune systems, which then control iron metabolism and contribute to an equilibrium competition pattern between the microbes and the host (Sangkhae and Nemeth, 2017). An increasing number of studies have revealed that iron deposition is implicated in many physiological and pathological processes of several diseases (Fei et al., 2024; Xu et al., 2023). Ferritin is the major intracellular iron storage protein, which exerts a significant function in regulating iron homeostasis (Orino and Watanabe, 2008; Wang et al., 2010). It is known that ferritin is highly expressed in the cytoplasm, nucleus, and mitochondrion (Beazley et al., 2009; Surguladze et al., 2005). Previous research studies have hinted that ferritin elevation is implicated in various cancers, acute infectious diseases, and inflammatory and autoimmune diseases (Kirkali et al., 1999; Ganz and Nemeth, 2009; Zandman-Goddard and Shoenfeld, 2007; Chen et al., 2020). Recent studies have revealed that the level of ferritin is closely related to the severity in coronavirus disease 2019 (COVID-19) (Zhou et al., 2020; Feld et al., 2020). A report from our laboratory has found that pulmonary ferritin is dramatically elevated in arsenic-incurred acute lung injury models (Li et al., 2022). Moreover, plasma ferritin can indicate macrophage activation-like syndrome and microbial etiology of CAP patients (Brands et al., 2021; Oppen et al., 2021). However, the concrete molecular mechanism and biological function of ferritin remained unclear in CAP patients.

So far, the exact evidence about the role of ferritin was absent in CAP. Moreover, the link of ferritin with CAP was also obscure. As a result, the goal was to estimate the relationship of serum ferritin with CAP. A prospective cohort study was designed and carried out. Eligible cases with severe CAP were recruited, and biological samples were obtained. Our results first provided evidence that ferritin might take part in the process of CAP.

## Materials and methods

### Subjects

According to the diagnostic criteria of CAP (Cao et al., 2018), CAP patients were selected from the Department of Respiratory and Critical Care Medicine in No. 2 People’s Hospital of Fuyang City and the Second Affiliated Hospital of Anhui Medical University. All CAP patients included must have had at least one of the following clinical symptoms: cough; expectoration; the number of white blood cells (WBC) was higher than 10 × 10^9^/L or less than 4 × 10^9^/L; radiographic detection found the characteristics of patchy, lobulated, and alveolar high-density infiltrating lesions; the temperature was up to 38.0°C; there were positive pathogen detection and moist rales; and partial cases may be accompanied with dyspnea. In addition, other examinations were conducted, and heart failure, organizing pneumonia, pulmonary embolism, tuberculosis, and other diseases were all eliminated in the current research. Finally, 273 patients confirmed with CAP were selected, and fasting blood samples were collected from CAP patients on admission before any intervention and treatment. Simultaneously, clinical characteristics and demographic information were obtained from all the participants. The inclusion criteria were as follows: conformed to the diagnostic criteria, older than 18 years, occurred in the community, participated in this research voluntarily, and there was no oral or intravenous iron treatment one month before hospitalization. The exclusion criteria were as follows: pregnant women; complicated with other pulmonary diseases; cancer, with hematological malignancies in particular; and complicated with immunodeficiency diseases, severe liver dysfunction, renal dysfunction, anemia, and malnutrition (Feng et al., 2021b; Zheng et al., 2021a; Xu et al., 2022). The severe and very severe patients were in the intensive care unit, and mild and moderate cases were in the general ward. We used CAP severity scores to evaluate pneumonia severity. Moreover, primal prognostic outcomes were tracked up and observed in the general ward or the intensive care unit during hospitalization (Cao et al., 2022; Liu et al., 2021). Finally, CAP cases with incomplete information, unavailable serum specimen, and without prognosis were all ruled out; 273 CAP subjects were recruited in this study (Supplementary Figure S1). In order to compare the level of serum ferritin, healthy volunteers who did not have a history of pulmonary disease were selected from the physical examination center. At last, sex- and age-matched healthy volunteers were included in this project. In addition, serum specimens were collected from partial patients before being discharged.

### Enzyme-linked immunosorbent assay (ELISA)

Analysis of ferritin was carried out in serum samples by ELISA. Ferritin (CSB-E05187h) commercial ELISA kits were bought from Cusabio (https://www.cusabio.com/). After hospitalization, peripheral blood samples were collected. Restricting the diet and water intake was necessary in CAP patients when we collected peripheral blood samples. Then, serum samples were obtained and stored in our biological sample bank (Fu et al., 2021; Fei et al., 2019). The concentration of ferritin was measured according to the manufacturer’s instructions with minor adjustments (Zheng et al., 2021b). Each standard or sample was detected in duplicate.

### Statistical analysis

All statistical analyses were conducted using SPSS 19.0 software. All continuous variables were represented as mean or median. The categorical variables were expressed as frequency. According to the tertiles of ferritin levels, CAP cases were divided into three groups. Demographics information, clinical characteristics, and laboratory data were compared *via* Student’s t-test, one-way ANOVA test, or the chi-square test among the three groups. The relationship of serum ferritin with clinical indicators was evaluated by performing the Spearman or Pearson correlative analysis. Linear and logistic regression models were used to establish the correlations of serum ferritin with CAP severity scores. Potential confounding factors were controlled. The relevancy of serum ferritin content with the prognosis was explored by the logistic regression model. Nonparametric receiver operating characteristic (ROC) curves of serum ferritin and other clinical parameters were constructed to discriminate the predictive powers. Statistical significance was defined as *p*-value < 0.05.

## Results

### Demographic information and laboratory data

Demographic information of all CAP patients was estimated. Demographic information and laboratory data are represented in Table 1. Among 540 CAP patients, the average age was 59 ± 18 years, and there were 162 (59.3%) male subjects. Moreover, the comorbidities were recorded in the participants. The number of cerebral infarctions was gradually elevated along with serum ferritin. In addition, routine blood indexes, liver function, renal function, and inflammatory cytokines were assessed. As represented in Table 1, the levels of white blood cell (WBC), neutrophil, aspartate aminotransferase (AST), urea nitrogen, creatinine, procalcitonin (PCT), D-dimer, C-reactive protein (CRP), and interleukin-6 (IL-6) were elevated with increasing serum ferritin. On the contrary, the count of lymphocyte gradually reduced in parallel with increasing serum ferritin (Table 1).

**TABLE 1:** Demographic information of 540 CAP patients.

Characteristic	All participators	Tertile of serum ferritin (ng/mL)			*P*
		T1 (<116.1)	T2 (116.1∼175.1)	T3 (>175.1)	
N	273	91	91	91	
Age (years)	59 ± 18	52 ± 19	58 ± 17	67 ± 14	**<0.001**
Male, n (%)	162 (59.3)	41 (45.1)	61 (67.0)	60 (65.9)	**0.004**
Heart rate (beats per min)	89 ± 18	87 ± 17	89 ± 19	92 ± 19	0.240
Respiratory rate (breaths per min)	20 ± 3	20 ± 5	19 ± 1	20 ± 3	0.098
Oxygen saturation (%)	94.7 ± 9.6	95.4 ± 7.4	95.9 ± 3.8	92.7 ± 14.3	0.054
Temperature (°C)	36.7 ± 0.7	36.7 ± 0.7	36.7 ± 0.6	36.7 ± 0.8	0.650
Systolic pressure (mmHg)	125.1 ± 19.3	123.8 ± 18.2	125.7 ± 18.2	125.8 ± 21.6	0.732
Diastolic pressure (mmHg)	75.1 ± 11.8	74.7 ± 11.2	75.1 ± 12.4	75.6 ± 11.8	0.867
Hypertension, n (%)	76 (27.8)	25 (27.5)	25 (27.5)	26 (28.6)	1.000
Diabetes mellitus, n (%)	30 (11.0)	8 (8.8)	10 (11.0)	12 (13.2)	0.397
Cerebral infarction, n (%)	26 (9.5)	9 (9.9)	2 (2.2)	15 (16.5)	**0.003**
Coronary heart disease, n (%)	14 (5.1)	2 (2.2)	7 (7.7)	5 (5.5)	0.277
Bronchitis, n (%)	3 (1.1)	2 (2.2)	0	1 (1.1)	0.775
White blood cell (10^9^/L)	8.2 ± 4.1	7.7 ± 4.2	7.7 ± 3.2	9.3 ± 4.5	**0.013**
Neutrophil (10^9^/L)	6.2 ± 3.9	5.6 ± 4.0	5.7 ± 3.1	7.5 ± 4.2	**0.001**
Lymphocyte (10^9^/L)	1.3 ± 0.8	1.5 ± 0.7	1.3 ± 1.0	1.0 ± 0.6	**0.001**
Alanine aminotransferase (U/L)	22.0 (13.0, 38.0)	20.0 (12.0, 34.0)	23.0 (12.0, 41.0)	25.0 (17.0, 41.0)	0.134
Aspartate aminotransferase (U/L)	24.0 (18.0, 36.5)	20.0 (16.0, 32.0)	26.0 (19.0, 35.0)	27.0 (19.0, 45.0)	**0.005**
Uric acid (μmol/L)	267.0 (191.5, 342.0)	277.0 (200.0, 331.0)	276.9 (210.0, 348.0)	243.0 (176.0, 336.0)	0.321
Urea nitrogen (mmol/L)	5.2 (4.0, 6.9)	5.0 (3.7, 6.3)	4.9 (3.9, 6.0)	6.3 (4.3, 9.1)	**<0.001**
Creatinine (μmol/L)	64.0 (50.0, 82.0)	60.0 (48.0, 79.0)	64.0 (52.0, 73.2)	70.0 (51.0, 89.0)	**0.030**
Procalcitonin (ng/L)	0.1 (0.03, 0.4)	0.04 (0.01, 0.2)	0.1 (0.03, 0.5)	0.3 (0.1, 0.7)	**<0.001**
D-dimer (mg/L)	0.8 (0.4, 2.0)	0.5 (0.3, 0.9)	0.7 (0.3, 1.5)	1.7 (0.8, 2.9)	**<0.001**
C-reactive protein (mg/L)	44.9 (7.5, 138.5)	12.7 (3.4, 70.2)	35.5 (7.4, 141.8)	113.6 (32.7, 168.5)	**<0.001**
Interleukin-6 (pg/mL)	34.6 (13.4, 56.3)	14.6 (4.5, 41.3)	36.7 (15.2, 56.3)	45.6 (29.6, 72.7)	**<0.001**

Bold values indicate statistical significance.

### Associations between serum ferritin and CAP severity scores

**FIGURE 1:**
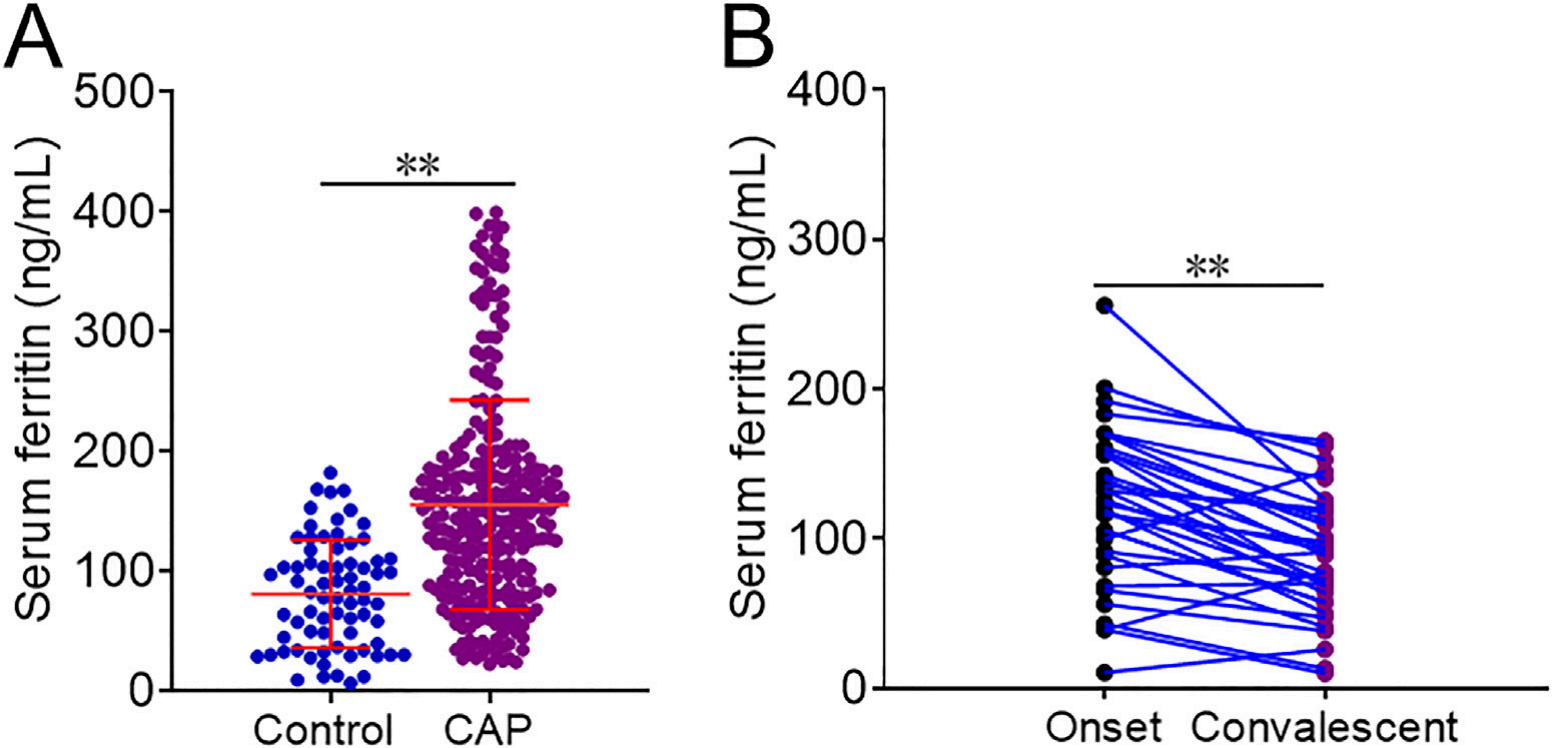
Levels of serum ferritin in different populations. **(A,B)** Levels of serum ferritin were detected and compared in different groups. **(A)** CAP patients versus control subjects. **(B)** CAP patients in the onset phase versus convalescent stage. **p < 0.01.

As represented in Figure 1A, the level of serum ferritin was markedly higher in CAP patients (156.1 ± 87.5 ng/mL) than in healthy volunteers (85.6 ± 6.6 ng/mL) (*p* < 0.01). Moreover, serum ferritin was decreased when the patients were discharged from hospital after therapy (122.1 ± 53.3 ng/mL versus 86.9 ± 40.8 ng/mL) (*p* < 0.01) (Figure 1B). Then, the associations of serum ferritin with the severity scores were analyzed. The potential confounding factors, such as age, gender, hypertension, diabetes mellitus, cerebral infarction, coronary heart disease, bronchitis, and diabetes mellitus, were controlled in the mixed logistic regression models. As shown in Table 2, linear aggression analysis found that each 1 ng/mL elevation of serum ferritin was related to score upregulations of 0.004 [(95% confidence interval (CI): 0.002∼0.005)], 0.003 (95% CI: 0.002∼0.004), 0.243 (95% CI: 0.206∼0.281), 0.007 (95% CI: 0.004∼0.009), and 0.029 (95% CI: 0.021∼0.036) in CURB-65, CRB-65, PSI, SMART-COP, and APACHE Ⅱ, respectively. In addition, logistic regression analysis indicated that serum ferritin concentration was positively correlated with CURB-65 (*p-*trend = 0.001), CRB-65 (*p-*trend = 0.002), and PSI (*p-*trend = 0.005) scores among CAP patients.

**TABLE 2:** Associations between serum ferritin and CAP severity scores.

Variables	Estimated changes by serum legumain (ng/mL)	Estimated changes (95% CI) by tertiles of serum ferritin (ng/mL)			*P* trend
		T1 (<113.7)	T2 (113.7∼159.4)	T3 (>159.4)	
N	293	91	91	91	
CURB-65	**0.004 (0.003, 0.005)**	1.0 (Ref)	1.195 (0.495, 2.889)	**4.194 (1.870, 9.405)**	**0.001**
CRB-65	**0.003 (0.002, 0.004)**	1.0 (Ref)	1.419 (0.542, 3.720)	**3.864 (1.597, 9.349)**	**0.002**
PSI	**0.243 (0.206, 0.281)**	1.0 (Ref)	**2.301 (1.047, 5.061)**	**3.576 (1.583, 8.077)**	**0.005**
SMART-COP	**0.007 (0.004, 0.009)**	1.0 (Ref)	0.631 (0.297, 1.341)	1.903 (0.947, 3.824)	0.059
APACHE Ⅱ	**0.029 (0.021, 0.036)**	1.0 (Ref)	0.452 (0.216, 0.945)	1.359 (0.664, 2.782)	0.360

Models were adjusted for age, gender, hypertension, diabetes mellitus, cerebral infarction, coronary heart disease, bronchitis, and diabetes mellitus. Bold values indicate statistical significance.

### Associations between serum ferritin and clinical parameters

**FIGURE 2:**
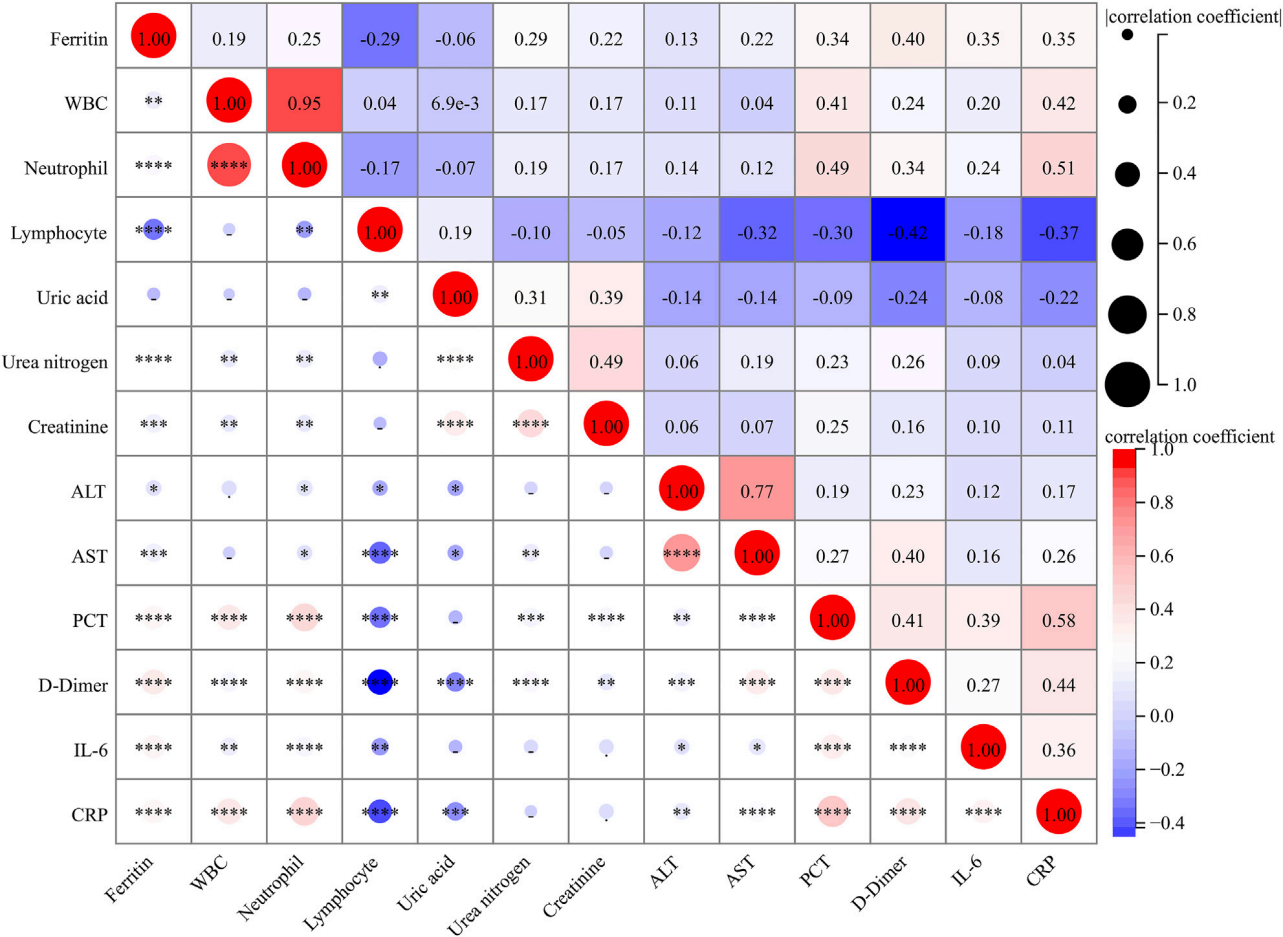
Correlations between serum ferritin and clinical parameters. The correlations of serum ferritin with clinical parameters were analyzed using Spearman correlation coefficient or Pearson rank correlation.

As represented in Figure 2, there were dramatic and positive correlations of serum ferritin with WBC (r = 0.191; *p* = 0.002) and neutrophil (r = 0.252; *p* < 0.001), and it was inversely associated with lymphocyte (r = −0.288; *p* < 0.001) in CAP patients. In addition, serum ferritin was positively and weakly related to urea nitrogen (r = 0.286; *p* < 0.001), creatinine (r = 0.223; *p* < 0.001), alanine aminotransferase (ALT) (r = 0.131; *p* = 0.030), AST (r = 0.215; *p* < 0.001), PCT (r = 0.343; *p* < 0.001), and D-dimer (r = 0.401; *p* < 0.001). In addition, the positive correlations of serum ferritin with inflammatory cytokines, including IL-6 (r = 0.350; *p* < 0.001) and CRP (r = 0.347; *p* < 0.001), were observed.

### Associations between serum ferritin and prognostic outcomes

During hospitalization, prognostic outcomes were tracked up and analyzed. As represented in Supplementary Figures S2A–C, the content of serum ferritin at admission was prominently elevated in cases with mechanical ventilation, vasoactive agent, and ICU admission compared with cases without the above prognostic outcomes. Furthermore, the content of serum ferritin was higher in dead patients than in surviving subjects (Supplementary Figures S2D). Additionally, serum ferritin was significantly increased in subjects with more than 14 hospital stays than in subjects with other hospital stays (Supplementary Figures S2E). Furthermore, compared with CAP patients in tertile 1, the numbers of CAP patients with mechanical ventilation, vasoactive agent usage, ICU admission, death, and longer hospital stays were evidently higher in tertile 3 (Table 3). Logistic regression analysis suggested that the relative risk (RR) of the poor prognosis was remarkably upregulated with elevated serum ferritin in CAP patients (Table 3).

**TABLE 3:** Adjusted relative risk for prognostic outcomes by tertiles of serum ferritin.

Variables	Tertiles of serum ferritin (ng/mL)			*P* trend
	T1 (<113.7)	T2 (113.7∼159.4)	T3 (>159.4)	
N	91	91	91	
Mechanical ventilation				
N (%)	7 (7.7)	6 (6.6)	32 (35.2)	**<0.001**
Unadjusted RR (95% CI)	Ref (1)	0.847 (0.273, 2.626)	**6.508 (2.692, 15.738)**	**<0.001**
Adjusted RR (95% CI)	Ref (1)	0.707 (0.202, 2.481)	**4.570 (1.663, 12.560)**	**<0.001**
Vasoactive agent				
N (%)	2 (2.2)	5 (5.5)	17 (18.7)	**<0.001**
Unadjusted RR (95% CI)	Ref (1)	2.587 (0.489, 13.694)	**10.223 (2.287, 45.691)**	**<0.001**
Adjusted RR (95% CI)	Ref (1)	1.578 (0.267, 9.319)	**6.963 (1.393, 34.808)**	**0.006**
ICU admission				
N (%)	8 (8.8)	7 (7.7)	31 (34.1)	**<0.001**
Unadjusted RR (95% CI)	Ref (1)	0.865 (0.300, 2.492)	**5.360 (2.302, 12.482)**	**<0.001**
Adjusted RR (95% CI)	Ref (1)	0.867 (0.260, 2.888)	**4.205 (1.539, 11.492)**	**0.001**
Death				
N (%)	1 (1.1)	2 (2.2)	18 (19.8)	**<0.001**
Unadjusted RR (95% CI)	Ref (1)	2.022 (0.180, 22.705)	**22.192 (2.894, 170.191)**	**<0.001**
Adjusted RR (95% CI)	Ref (1)	1.417 (0.119, 16.930)	**16.572 (2.004, 137.033)**	**<0.001**
Longer hospital stays				
N (%)	12 (13.2)	15 (16.5)	32 (35.2)	**0.001**
Unadjusted RR (95% CI)	Ref (1)	1.299 (0.571, 2.956)	**3.571 (1.696, 7.516)**	**<0.001**
Adjusted RR (95% CI)	Ref (1)	1.308 (0.540, 3.166)	**2.457 (1.083, 5.576)**	**0.025**

RR: relative risk. The length of hospital stay was divided into two groups: longer hospital stays, ≥14 days; lower hospital stays, <14 days. Models were adjusted for age, gender, hypertension, diabetes mellitus, cerebral infarction, coronary heart disease, bronchitis, and diabetes mellitus. Bold values indicate statistical significance.

### Predictive capacities for prognostic outcomes

**FIGURE 3:**
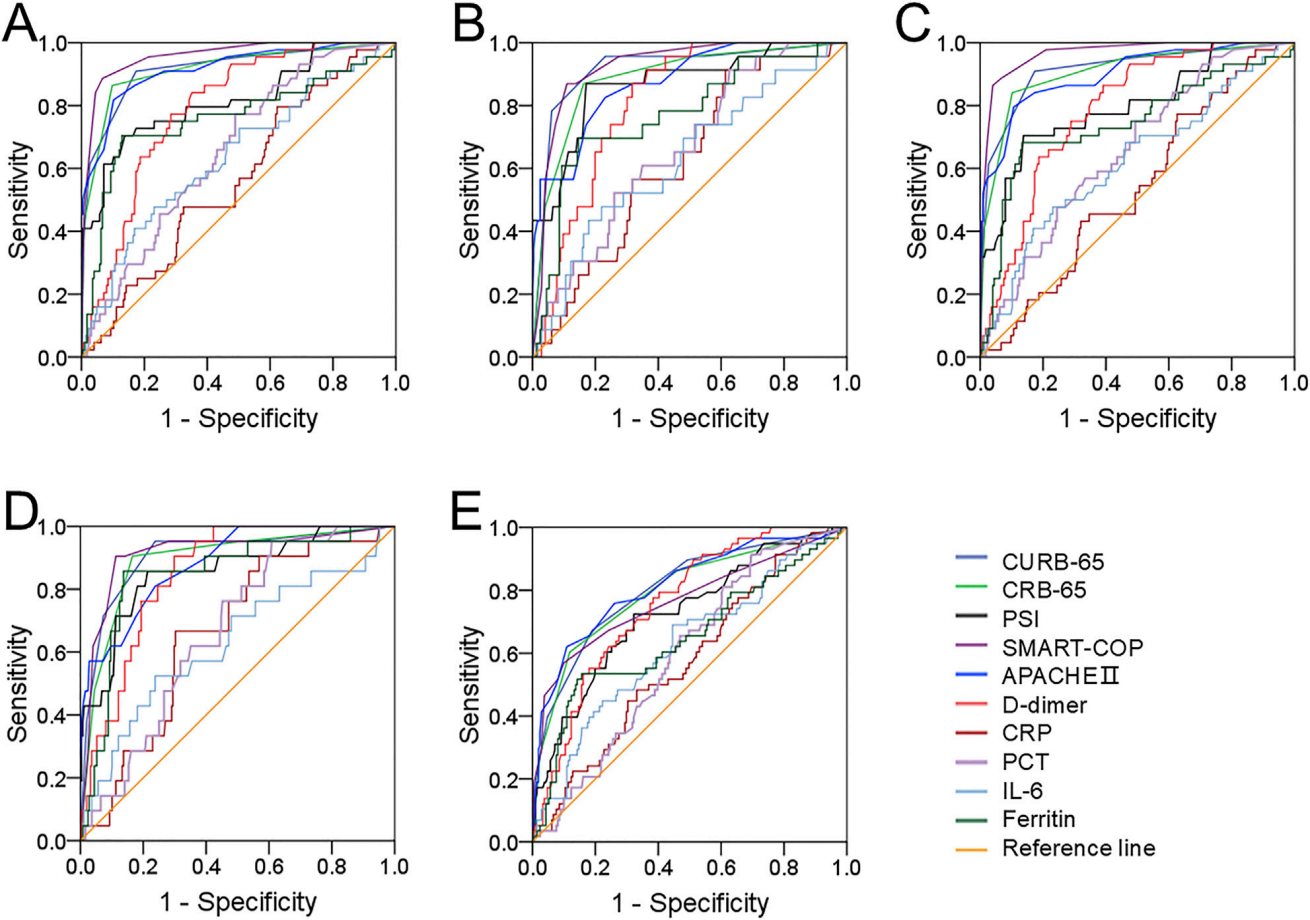
Predictive capacities for prognostic outcomes. **(A–E)** Predictive powers of different indicators for prognostic outcomes were evaluated in CAP patients through ROC curve. **(A)** Mechanical ventilation. **(B)** Vasoactive agent. **(C)** ICU admission. **(D)** Death. **(E)** Longer hospital stays prediction.

Predictive capacities of different clinical parameters for prognostic outcomes were evaluated through ROC area under the curve (AUC). We found that the distinguishing capacities for mechanical ventilation, vasoactive agent usage, and ICU admission were similar between serum ferritin and CAP severity scores, and they were higher than PCT, IL-6, CRP, and D-dimer (Figure 3). In addition, the predictive power for death was elevated in serum ferritin compared with other CAP severity scores and inflammatory cytokines (Figure 3). The cutoff level of ferritin was 203.8 ng/mL, and it had 85.7% sensitivity and 71.8% specificity for death prediction. On the contrary, the predictive efficiency of serum ferritin for longer hospital stay was lower than those of CAP severity scores, and it was similar with blood routine indexes, such as PCT, D-dimer, IL-6, and CRP (Figure 3).

## Discussion

In the current investigation, CAP cases were enrolled in the study, and the level of serum ferritin was analyzed. We then explored the diagnostic and predictive powers of serum ferritin for CAP patients *via* a prospective cohort. We found that the concentration of serum ferritin was prominently upregulated in CAP patients compared with healthy volunteers. In addition, there were obvious associations between serum ferritin at admission and CAP severity scores. Moreover, serum ferritin was strongly related to several parameters. In addition, serum ferritin at admission was positively associated with poor prognosis. Not only that but also there was a higher predictive power for death in serum ferritin than those in other severity scores and blood routine indices.

Ferritin is a ubiquitously expressed spherical protein and is the major intracellular iron storage protein, which regulates iron homeostasis (Wang et al., 2010; Torti and Torti, 2002). Previous research studies have shown that ferritin is involved in the pathogenesis of various inflammatory diseases. Increasing ferritin contents are associated with acute or chronic inflammatory conditions whether evoked by infections or not (Sharif et al., 2018; Ueda and Takasawa, 2018). A retrospective longitudinal analysis found higher ferritin level in COVID-19 patients (Qeadan, et al., 2021). Additionally, the initial serum ferritin is upregulated in acute respiratory distress syndrome (ARDS) (Sharkey, et al., 1999). In addition, the expression of ferritin is increased and inversely associated with the pulmonary function level in chronic obstructive pulmonary disease (COPD) (Zhang et al., 2020). Nevertheless, the evidence about the role of ferritin in CAP remained lacking. Therefore, we measured the serum ferritin level in the participants. Our results found that the initial serum ferritin level was increased in CAP patients compared with healthy volunteers, and it gradually upregulated with increased CAP severity scores. Correlative analysis unveiled that the initial serum ferritin level was strongly associated with many clinical indicators. Moreover, regression analysis confirmed a positive relationship of the serum ferritin level with CAP severity scores. So, it clearly stated that ferritin participated in the pathogenesis of CAP.

Cumulative proof suggested that ferritin expression is closely related to many prognostic outcomes in lots of diseases. An earlier report indicated that the initial serum ferritin content is notably increased in COVID-19 patients with severe acute liver injury, ICU transfer, and mechanical ventilation (Cheng et al., 2020). Moreover, higher serum ferritin content elevates the risk of metabolic syndrome throughout childhood (Suárez-Ortegón et al., 2019). Higher expression of ferritin mRNA in kidney is positively correlated with metastasis and bad prognosis in renal cell carcinoma (Huang et al., 2019). Interestingly, serum ferritin expression at admission was increased in CAP patients, which occurred with various poor prognoses. Then, the logistic regression model showed that the serum ferritin level at admission was positively linked to poor prognosis. Moreover, ROC curve analysis indicated that the discriminability for death was considerably higher in serum ferritin than in CAP severity scores and blood routine indices. In short, these data provided evidence that higher serum ferritin at admission increases the incidence of bad prognosis in CAP patients during hospitalization.

The mechanism of ferritin upregulation was obscure in CAP. Under normal physiological conditions, ferritin synthesis is regulated by and relies on the iron content. Several animal experiments showed that ferritin secretion is evoked through elevating serum iron levels (Zhang et al., 2021). Not only that, many studies stated that ferritin synthesis is also regulated by inflammation, oxidative stress, growth factors, hypoxia–ischemia, and hyperoxia (Koorts and Viljoen, 2007). Moreover, ferritin is one of the downstream target genes of several transcription factors, including NF-κB and Nrf-2. Research found that NF-κB can bind to DNA elements of ferritin promoter and regulate ferritin expression (Torti and Torti, 2002). In addition, Nrf-2 mediates transcriptional activation of ferritin and upregulates ferritin expression by combining with the binding site of electrophile/antioxidant responsive element (EpRE/ARE) (Pietsch et al., 2003). Due to the inflammatory and infectious disease (CAP), Nrf-2 and NF-κB are activated in the animal models of pneumonia (LaCanna et al., 2019; Sun et al., 2020). Several inflammatory cytokines can elicit the release of ferritin into the blood stream (Fahmy and Young, 1993). Therefore, we thought that different pathogenic microbes’ infection may evoke an inflammatory reaction and activate nuclear transcription factors, such as NF-κB and Nrf-2, in pulmonary epithelial cells. At last, the transcriptional expression of ferritin is upregulated in CAP.

This investigation found that serum ferritin concentration was positively associated with the severity scores and poor prognosis *via* a prospective cohort study. These results hinted that serum ferritin can predict the risk of poor prognosis among CAP patients in advance. In addition, the clinician can take intervention measures to avoid the adverse progress ahead, such as ICU admission, mechanical ventilation, noninvasive positive pressure ventilation, and vasoactive agent usage, to improve the prognosis. However, this study was only an observational study, and the potential mechanism of ferritin elevation was ambiguous. The effect of ferritin inhibition on the progression of CAP was unclear. We did not know whether ferritin expression can be used as a therapeutic target for CAP. In the future, more animal and cellular experiments can solve these doubts. Therefore, for the abovementioned reasons, we thought that serum ferritin can be used a biomarker to estimate the severity and adverse prognosis in CAP patients. Although there were some advantages of this study, we must also recognize the inevitable limitations. First, this investigation was a single-center study with a small sample size; a multicenter study with a large sample size is needed to further confirm the current conclusion. Second, this study only detected the level of ferritin in serum, and the local expression of ferritin cannot be evaluated.

## Conclusion

This prospective cohort study assessed the relationships of the serum ferritin level with severity and prognosis. Our data suggested that serum ferritin is upregulated in CAP cases compared with control subjects. Additionally, the serum ferritin level at admission is strongly and prominently related to the disease severity and prognosis. Furthermore, serum ferritin has a higher predictive power for death than CAP severity scores and blood routine indices. These results provide evidence that ferritin may participate in the process of CAP, but the target cells and specific mechanisms remain to be illuminated. Consequently, more experiments should be conducted to understand the function of ferritin elevation and ascertain the therapeutic role of anti-ferritin treatment in CAP patients.

## Data Availability

The original contributions presented in the study are included in the article/Supplementary Material. Further inquiries can be directed to the corresponding authors.
