# Supplementary material for: Longitudinal relationships between serum ferritin and prognosis among severe community-acquired pneumonia patients
Source: Front Pharmacol. 2025 May 20;16:1556185. doi: 10.3389/fphar.2025.1556185 (PMC12131277; doi:10.3389/fphar.2025.1556185)
Supplement: Supplementary file 1 [file DataSheet1.doc]

**Supplemental Figures**


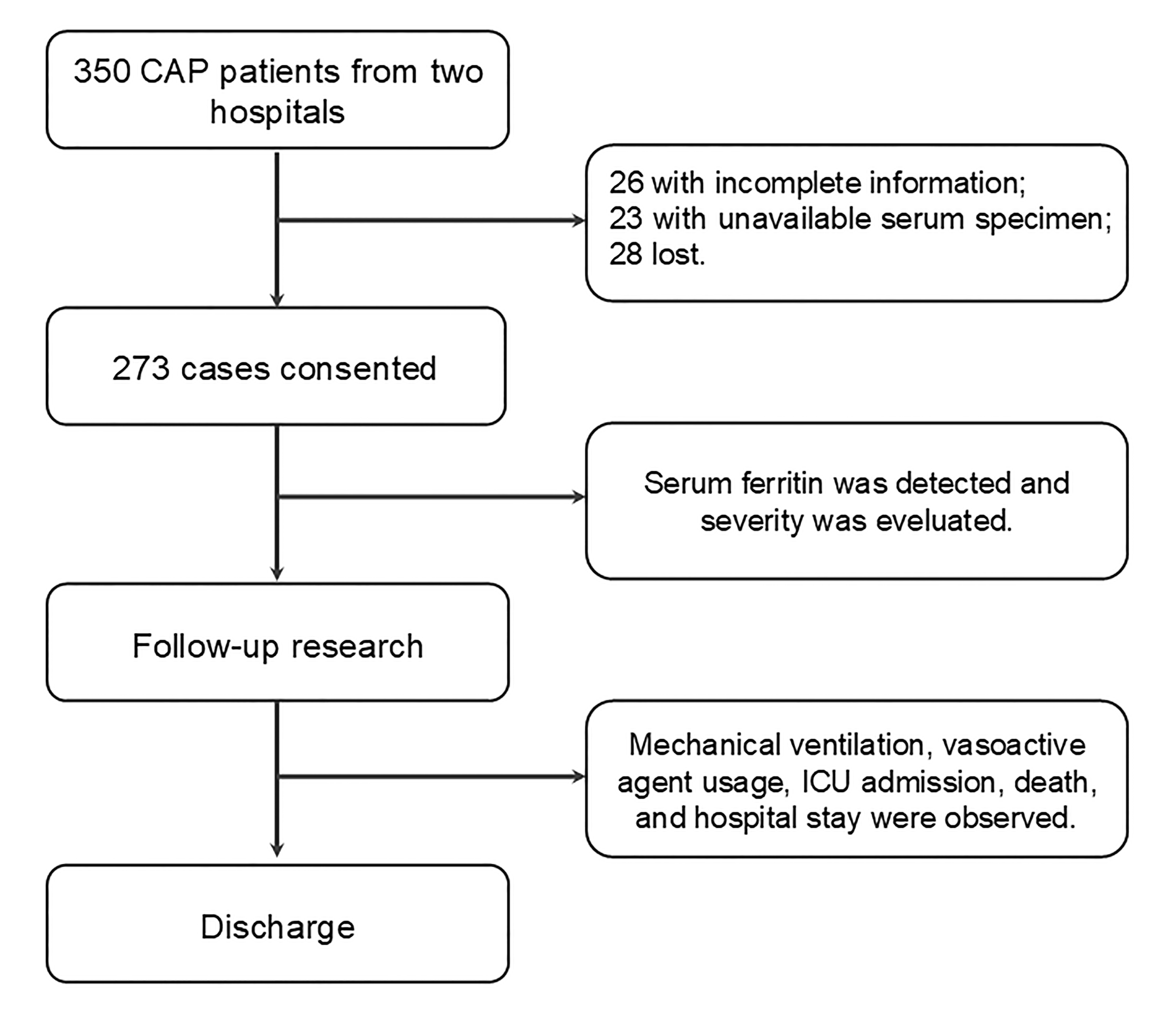


**Supplemental Figure 1. Flow diagram of recruitment and follow-up research.**


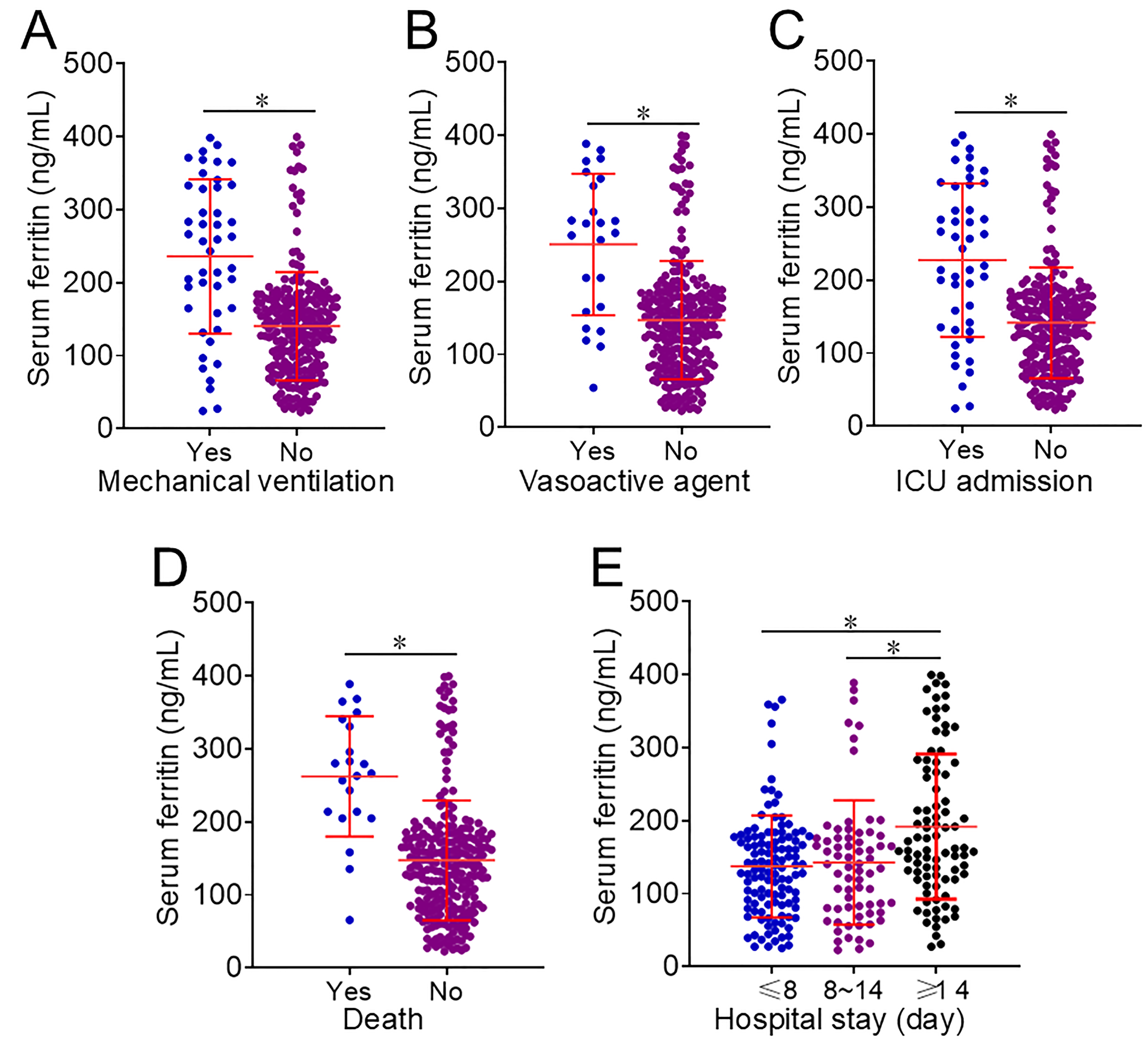


**Supplemental Figure 2. The levels of serum ferritin in CAP patients with different prognostic outcomes.**

(A-E) The levels of serum ferritin were detected in CAP patients with different prognostic outcomes through ELISA. (A) The levels of serum ferritin were detected in CAP patients with or without mechanical ventilation. (B) The levels of serum ferritin were detected in CAP patients with or without vasoactive agent usage. (C) The levels of serum ferritin were detected in CAP patients with or without ICU admission. (D) The levels of serum ferritin were detected in CAP patients with or without death. (E) The levels of serum ferritin were detected in CAP patients with or without vasoactive agent usage. The statistical difference was analyzed by student's t tests. **P*＜0.05.
